# Practical Urine Testing

**Published:** 1887-10

**Authors:** 


Practical Urine Testing, a guide to Office and Bedside Urine Analysis, for Physicians and Students, by Charles Godwin Jennings, M. D., Prof. Chemistry and Diseases of Children, Detroit Medical College, Detroit, D. O. Hynes & Co., 1887, cloth bound, p. 124.

The real importance of a knowledge of the constituents of the urine in disease, and of their significance, is scarcely appreciated by the majority of practitioners; but the more advanced understand the value of such an acquaintance; and now, it is almost a universal practice in the graver diseases to analyse the Urine. It is a cause for congratulation that the process has been so much simplified. All Life Insurance Companies require it. The book before us is a valuable guide to the inexperienced, and of value also, to the more expert. It contains, in brief, but explicit terms, description of the physical characteristics of normal and of abnormal urine, details the processes by which both quantative and qualitative analyses are made; gives microscopic examinations, apparatus and reagents, analyses of calculi, etc., the meaning of the presence of such and such elements, etc.; a valuable little companion which any one can study with profit.

